# Sensitive and Reproducible Gold SERS Sensor Based on Interference Lithography and Electrophoretic Deposition

**DOI:** 10.3390/s18114076

**Published:** 2018-11-21

**Authors:** June Sik Hwang, Minyang Yang

**Affiliations:** Department of Mechanical Engineering, Korea Advanced Institute of Science and Technology, Daejeon 34141, Korea; ssvsjs76@kaist.ac.kr

**Keywords:** surface enhanced Raman spectroscopy, gold nanoparticle array, laser interference lithography, electrophoretic deposition, pulsed laser ablation in liquid

## Abstract

Surface-enhanced Raman spectroscopy (SERS) is a promising analytical tool due to its label-free detection ability and superior sensitivity, which enable the detection of single molecules. Since its sensitivity is highly dependent on localized surface plasmon resonance, various methods have been applied for electric field-enhanced metal nanostructures. Despite the intensive research on practical applications of SERS, fabricating a sensitive and reproducible SERS sensor using a simple and low-cost process remains a challenge. Here, we report a simple strategy to produce a large-scale gold nanoparticle array based on laser interference lithography and the electrophoretic deposition of gold nanoparticles, generated through a pulsed laser ablation in liquid process. The fabricated gold nanoparticle array produced a sensitive, reproducible SERS signal, which allowed Rhodamine 6G to be detected at a concentration as low as 10^−8^ M, with an enhancement factor of 1.25 × 10^5^. This advantageous fabrication strategy is expected to enable practical SERS applications.

## 1. Introduction

Surface-enhanced Raman spectroscopy (SERS) has attracted a significant amount of attention in chemical [1,2] and biological [3,4,5] analyses because it can detect a molecule’s specific fingerprint with high sensitivity, which enables the detection of even a single molecule [6]. A highly enhanced Raman signal, which allows for this superior SERS sensitivity, is mainly attributed to the increased electromagnetic fields through localized surface plasmon resonance (LSPR). Since the Raman signal was found to be dramatically enhanced by hot spots in adjacent nanostructures from the LSPR [7], various metallic nanostructures with rough surfaces have been studied.

A bottom-up approach based on noble metal colloids is mostly used for SERS substrates due to its simple configuration and tunable surface plasmon resonance (SPR). In addition to the different sizes [8] and shapes [9,10,11] of metal nanoparticles (NPs), self-assembly [12], electroless deposition [13], and other strategies [14,15,16] have been reported. These methods are highly sensitive, simple, and inexpensive, but they often have low reproducibility owing to the randomly deposited NPs [17]. 

Several top-down methods, which assure the high sensitivity and reproducibility of SERS substrates, have also been studied, including e-beam lithography [18], the focused ion beam (FIB) process [19], nanosphere lithography [20], and nanoimprint lithography [21]. However, these approaches have limitations, such as low productivity, high complexity, expensive, low pattern variability, and high-cost mold fabrication. Therefore, a sensitive and reproducible SERS sensor based on a simple and inexpensive process is required.

Among the various SERS substrates, the periodic metal nanostructure has advantages: it has a reproducible signal, and its period and diameter can be controlled, enhancing the LSPR [22]. For this structure, we used laser interference lithography (LIL), which is a large-scale, simple, and low-cost process that can be performed in ambient conditions. In the LIL configuration, a lift-off process is usually used, which consists of photoresist (PR) template generation, metal deposition, and PR etching. LIL is a simple method for PR template fabrication, although metal deposition should also be considered when this process is used for the fabrication of an SERS sensor, because, for practical SERS applications, the whole fabrication procedure should be simple and inexpensive. The deposition of the target metal onto the PR template is mostly conducted via vacuum-based deposition, such as thermal deposition [23], physical vapor deposition (PVD) [24], and sputtering [25], which guarantee an excellent surface quality. However, these processes are complex and time-consuming, often reducing the advantages of LIL, which is a simple ambient-condition process. To overcome these disadvantages, several studies focused on the solution process, including the electroplating [26], spin coating [27], and self-assembly [28] of NPs. However, these methods have shortcomings, such as a smooth surface from atomic-scale deposition and requiring high-temperature post-processing to remove surfactant and ligands. In this regard, a simple metal deposition process is still needed.

The electrophoretic deposition (EPD) of NPs from pulsed laser ablation in liquid (PLAL) is a simple eco-friendly process that produces a pure metal surface, which has a higher grafting density than a chemical synthesized surface [29]. PLAL is a process that physically synthesizes NPs with bulk metal. When the optical energy above a threshold is irradiated on the target material, the material experiences plasma formation; following this, a cavitation bubble appears. Due to the strong influence of the cavitation bubble, the plasma plume is fragmented. As a result, condensed plumes become NPs [30]. Due to the physical synthesis, NPs formed via PLAL do not retain any surfactants or ligands and do not require high-temperature post-processing to remove by-products [31]. From the naturally generated zeta potential of these NPs, EPD can be applied for various metal nanostructures [32,33,34,35].

Herein, we report a simple processing method for fabricating a large-scale gold (Au) NP array based on LIL and EPD of Au NP from PLAL. The parameters of LIL, PLAL, and EPD were periodically adjusted to fabricate a roughened metal structure under various conditions. The sensitivity and reproducibility of the SERS substrate were examined using a well-known Raman probe, Rhodamine 6G (R6G). Given the advantages of this ambient, large-scale, simple, and inexpensive process, this fabrication strategy is expected to facilitate practical SERS applications in the real world.

## 2. Materials and Methods

**Figure 1:**
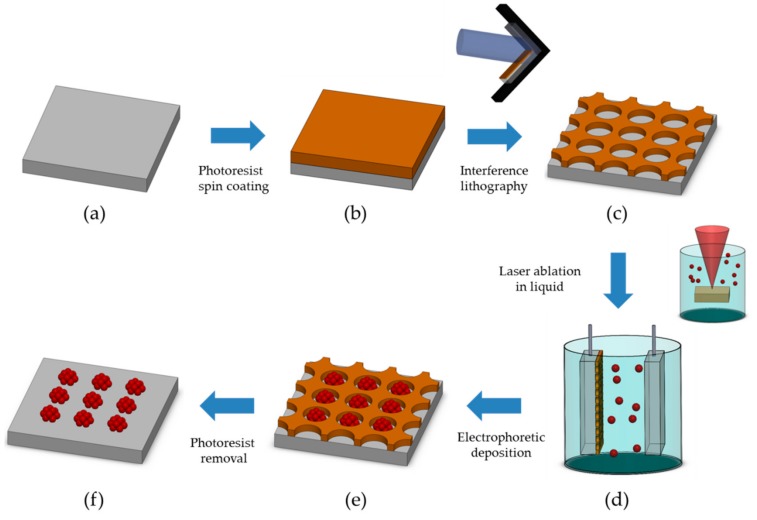
Schematic diagram of the fabrication process of the gold (Au) nanoparticle (NP) array: (**a**) a pristine electrode, (**b**) spin-coated photoresist (PR) layer on the electrode, (**c**) the PR nanohole patterned layer by laser interference lithography, (**d**) electrophoretic deposition (EPD) of Au NPs, (**e**) deposited Au NPs in the PR template, and (**f**) PR etching to form the Au NP array.

The whole experiment was conducted as shown in Figure 1. A periodic Au NP array was fabricated using LIL and EPD of Au NPs, generated from PLAL. At first, the spin coating of the photoresist on the indium tin oxide (ITO) substrate was conducted, and LIL was conducted. After a second exposure, a PR nanohole template on ITO was made, as shown in Figure 1c. Au NPs were produced using PLAL, which needs no surfactant or ligand for dispersion as they have a naturally-generated zeta potential. The EPD configuration for the deposition of Au NPs on the PR template is shown in Figure 1d. The ITO with PR templates and a pristine ITO were used as anode and cathode, respectively, because the generated Au NPs have a negative zeta potential. After the anodic EPD of Au NPs onto the PR template, and following PR etching, a periodic Au NP array with a rough surface was fabricated with a simple, low-cost, and ambient-condition process.

### 2.1. PR Nanohole Patterning on Electrodes through LIL

A Lloyd mirror interference system, in which it is easier to align optical components than in a multi-beam interference system, was used for PR nanohole patterning. An ultraviolet (355 nm) laser (10 ns, 20 kHz, 1 W, Spectra Physic, Santa Clara, CA, USA) was utilized for the suitable absorption of i-line PR. The scheme and a digital Lloyd mirror interference lithography image are shown in Figure S1a,b, respectively. The optical system first allowed the beam from the light source to pass through only an s-polarized beam using a polarized beam splitter (10 APF, Altechna, Vilnius, Lithuania). At this time, the intensity of the s-polarized beam was adjusted to be the largest, and the remaining p-polarized beam was blocked by a beam dump. The polarized beam was adjusted to obtain a clean Gaussian beam using a spatial filter consisting of a plano-convex lens (LA4725-UV-ML, focal length f = 75.0 mm, Thorlabs, Newton, MA, USA) and a 10-μm pinhole (P10D, Thorlabs, Newton, MA, USA). The distance between the lens and the pinhole was controlled by the focal length, so that the focused beam passed through the pinhole. After this beam passed a beam expander (6-BE-5X-355-S, Altechna, Vilnius, Lithuania), a collimated beam 11 mm in diameter was obtained. Before irradiation of the PR coated substrate, the exposure dose was measured and fixed at 0.31 mJ/cm^2^. An automatic shutter (SH05, Thorlabs, Newton, MA, USA) was also applied for a precise exposure time in order to supply a constant irradiation energy to substrates. The incident beam and a reflected beam from the mirror, perpendicular to the substrate, were adjusted to be overlaid to create the interference effect. In the configured Lloyd mirror system, the period of the hole could be adjusted by means of Equation (1), and the hole size could be controlled according to the exposure time. In our experiment, the angle between the incident beam and mirror was adjusted for the required period size, and the exposed PR formed a line pattern. For a nanohole pattern, a second exposure to the substrate, rotated by 90°, was conducted, and a periodic PR nanohole array was formed on the ITO electrode with an area of 2 × 2 mm^2^.

(1) $$\Lambda =\frac{\lambda }{2sin\theta }$$

where Λ is the period, λ is the wavelength of the incident light, and θ is the angle between the incident beam and the mirror.

The exposure was conducted following the typical process. An ITO (15 Ω/sq, All for Lab, Seoul, Korea), with an area of 12.7 × 12.7 mm^2^, was first ultrasonically cleaned in ethanol and deionized (DI) water for 5 min. For better adhesion during the EPD, a surface promoter (SURPASS 4000, Dischem, Midrand, South Africa) was spin-coated on ITO at 3000 rpm for 30 s and rinsed with DI water. The spin coating of positive PR (KL5302, KemLab, Woburn, MA, USA) was subsequently conducted at 3000 rpm for 60 s and a soft bake was conducted at 105 °C for 60 s. After 1 min of cooling, the collimated beam was irradiated on the substrate at a dose of 0.31 mJ/cm^2^ for 4 s. After the second exposure to the substrate, rotated by 90°, a post-exposure bake was conducted at 115 °C for 60 s, and then cooled for 1 min. Lastly, tetramethylammonium hydroxide (TMAH) developer solution was drop-casted to the exposed substrate for 20 s. After puddle-type development, the substrate was rinsed with DI water, followed by N_2_ blowing.

### 2.2. Preparation of Au Colloidal Solution

The Au colloidal solution was prepared using the PLAL of an Au plate (99.9%, MADELAB, Seoul, Korea) immersed in DI water (high performance liquid chromatography (HPLC) grade, Honeywell, Morris Plains, NJ, USA). The gold plate was polished by a polishing machine (Rotopol-2, Struers, Cleveland, OH, USA) to be less affected by the surface condition. As water was inserted, sandpaper (1200 CW 2C, DAE SUNG ABRASIVE Co., Seoul, Korea) was rotated at 300 rpm to grind the substrate. After 1 min of grinding, the gold plate was ultrasonically cleaned in ethanol and DI water for 5 min. After that, the Au plate was immersed in 30 mL of DI water and located 3 mm below the surface of the aqueous solution. Our PLAL process was simple, as shown in Figure S1c. An ytterbium fiber laser, with a wavelength of 1070 nm (YLM-10W, IPG Photonics, Oxford, MS, USA), was focused on a 75-μm spot using an f-theta lens, with a focal length of 254 mm, and a two-dimensional galvanometer scanner (SCANcube III 10-406987, SCANLAB, Puchheim, Germany). While the solution was constantly stirred at 300 rpm, the metal plate was irradiated for 30 s at a fluence of 7.55 J/cm^2^. During the process, continuous sparks and cracking sounds were generated from the gold plasma. After irradiation, a pinkish-colored Au NP solution formed. The concentration of the colloidal solution was estimated to be about 59 μg/mL from the mass loss from the Au plate.

### 2.3. EPD of Au NP 

With the fabricated PR hole-patterned ITO electrode and Au NPs generated from PLAL, EPD was conducted. A potentiostat (Vertex, IVIUM, Eindhoven, Netherlands) was used to apply a constant electric field, which was controlled by varying the voltage and distance between the substrates. During the process, PR-patterned ITO and pristine ITO were used as the anode and cathode, respectively. After two electrodes, with half the ITO area (6.35 × 12.7 mm^2^), were immersed in Au NP solution, 5 V/cm and 10 V/cm were applied, depending on the EPD time. After the EPD, PR-patterned ITO was rinsed with acetone and DI water followed by N_2_ blowing. At this time, Au NPs were deposited on an area of 2 × 2 mm^2^ forming a Au NP array.

### 2.4. Characterization

The surface morphologies of the samples were examined with a scanning electron microscope (NOVA 230, FEI, Hillsboro, OR, USA). The generated nanoparticles’ shape and size distribution were examined using a transmission electronic microscope (Tecnai F20, FEI, Hillsboro, OR, USA), followed by ImageJ processing. Component analysis was also performed by energy dispersive spectrometer (EDS). The absorbance and zeta potential were measured by a ultraviolet-visible (UV-Vis) spectrometer (Evolution 220, Thermo Fisher Scientific, Waltham, MA, USA) and Zetasizer Nano ZS (Malvern Panalytical, Westborough, MA, USA), respectively. 

For the SERS effect measurement, Rhodamine 6G (Sigma Aldrich, Saint Louis, MO, USA), which is a well-known Raman probe, was used. Drop casting of 10 µL Rhodamine 6G (R6G) solution on the SERS substrate was conducted, and naturally dried samples were used for Raman measurement. The Raman spectra of samples were obtained from an ARAMIS micro Raman spectrometer (Horiba Jobin Yvon, Kyoto, Japan), with a 514 nm laser source, 3 s acquisition time, and single integration. The measurement was conducted with a 0.35 mW laser power in both normal Raman and SERS. A 50× objective lens with 0.5 N.A. and 1 μm spot size was used for all Raman measurements. All Raman data were background-subtracted, and the baseline correction was conducted with fifth-order polynomial fitting.

## 3. Results and Discussion

**Figure 2:**
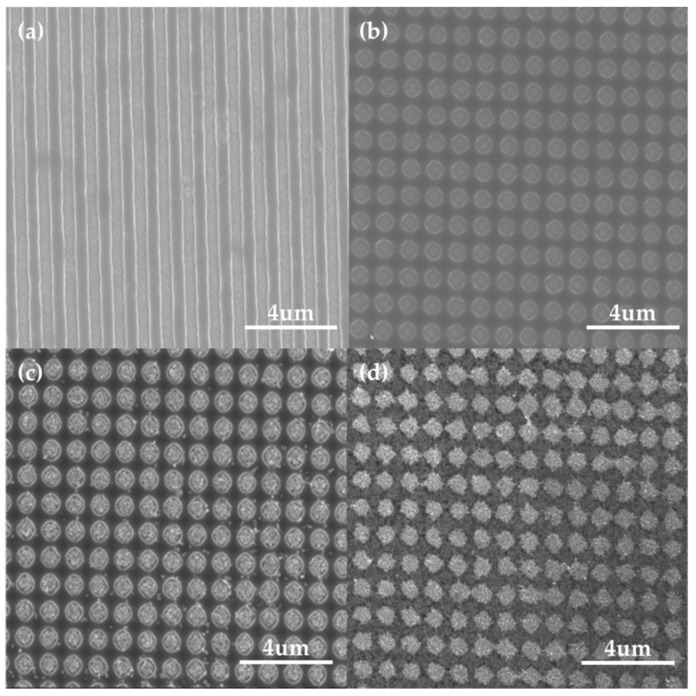
Scanning electron microscopy (SEM) image of (**a**) the PR line pattern and (**b**) the PR hole pattern on the indium tin oxide (ITO) and SEM images of the substrate, following (**c**) Au NP EPD and (**d**) PR etching.

The Au NP array fabrication process, combining a PR-patterned template with LIL and EPD of the Au NP from PLAL, is shown in Figure 2. The substrate produced by the first exposure generated a line pattern, with a 1-μm period in a 2 × 2 mm^2^ area (Figure 2a). The twice-exposed sample formed a nanohole array with a diameter of 808 nm, as shown in Figure 2b. After the EPD of the Au NP solution, we found that the Au NPs were well-deposited on the PR template. Finally, a PR-etched substrate formed a uniform Au NP array with good adhesion (Figure 2d). The atomic force microscopy (AFM) measurement showed that a periodically rough structure of gold bumps, with a height around 20 nm (Figure S2), was fabricated.

### 3.1. Characterization of the Au NP Produced by PLAL

In our PLAL process, the most important parameters for the colloidal size and dispersion were the fluence of the laser beam and the concentration of the solution. When too much energy, such as a fluence of 22.64 J/cm^2^, was irradiated on the target, a larger size distribution formed because the aggregation of the NPs increased as the ablated volume increased (Figure S3a). An excessive processing time for a high-concentration solution caused several disadvantages, including an increased temperature and partial evaporation of the aqueous solution and severe coagulation due to the collision with the adjacent nanoparticles. Thus, during the ablation process, the fluence and concentration were properly adjusted to 7.55 J/cm^2^ and around 59 μg/mL, respectively.

**Figure 3:**
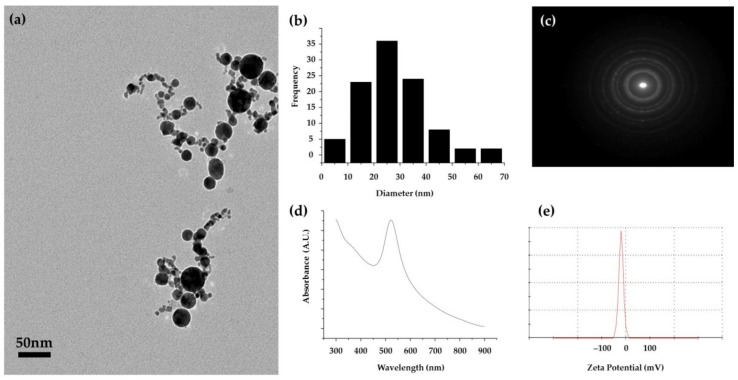
(**a**) Transmission electron microscopy (TEM) image, (**b**) size distribution, (**c**) selected area electron diffraction (SAED) pattern, (**d**) absorbance, and (**e**) zeta potential of the Au NP solution from PLAL.

To characterize the NPs synthesized by PLAL, the shape and size distributions of particles were measured using TEM. Some particles were aggregated, although the generated NPs were generally spherical and were less than 100 nm in size (Figure 3a). To identify the distribution, 100 samples were measured using TEM and processed through ImageJ. The mean size of the nanoparticles was 27 nm, and the standard deviation was 11 nm, which is a rather broad size distribution, using physical synthesis [36]. We measured the selective area electron diffraction (SAED) to determine the crystallinity of the generated nanoparticles, and this was consistent with the typical face-centered cubic (FCC) structure of gold (Figure 3c). The EDS measurement, shown in Figure S3b, helped to confirm the formation of gold nanoparticles. The solution had an absorbance peak at 522 nm, which is identical to the plasmon resonance absorption peak of gold. Au NPs, generated from PLAL, are known to have negative zeta potential due to the adsorption of –OH, which is dissipated during the laser ablation process [37]. The generated solution had a zeta potential of −10 mV, which allows for anodic EPD (Figure 3e). 

### 3.2. EPD of Au NP

Since it is important for reproducible SERS sensors to construct a nanostructure that has a regular pattern, suitable EPD conditions must be selected. In the EPD process, the yield of deposition can be simplified with the equation $\frac{dY}{dt}=\mu \times E\times S\times c$, where *Y* is the yield of deposition, *t* is the deposition time, μ is the electrophoretic mobility, *E* is the electric field, *S* is the surface area of the electrode, and *c* is the solid concentration [38]. In our case, electric mobility was usually determined according to the property of the gold colloidal solution. The deposition area was fixed to the exposed area. Additionally, the concentration was limited to an adequate level for the dispersion of Au NPs, because too-high of a concentration could induce the aggregation of nanoparticles during the PLAL process. Thus, the surface morphology, depending on the electric field and time, was examined to determine the proper EPD condition.

**Figure 4:**
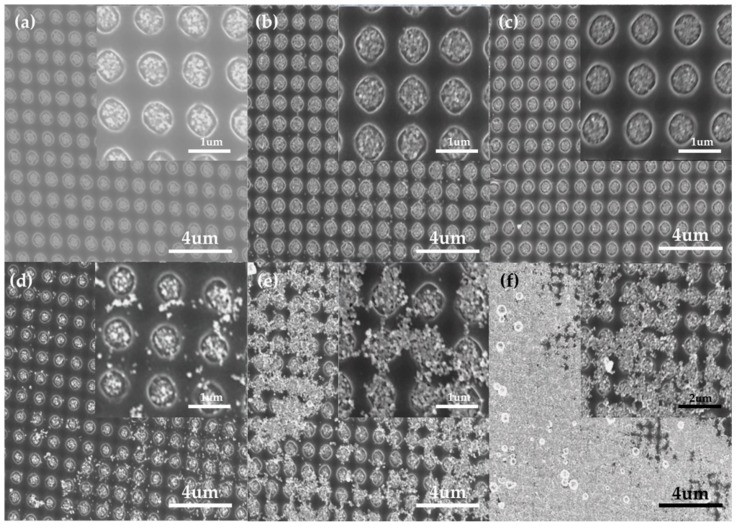
SEM image of the substrate after Au NP EPD: 5 V/cm with (**a**) 10 min, (**b**) 30 min, and (**c**) 60 min; and 10 V/cm with (**d**) 10 min, (**e**) 30 min, and (**f**) 60 min. Insets: The corresponding local magnification.

The surface characteristics of the fabricated substrates, depending on the electric field and EPD time, are shown in Figure 4. Under a low electric field, such as 5 V/cm, we observed that the nanoparticles were well-deposited in the PR template, forming a periodic and uniform gold nanoparticle structure (Figure 4a). Under a high electric field condition of 10 V/cm, the nanoparticle-connected structure appeared due to the excessive deposition of nanoparticles, as shown in Figure 4d. The over-formed deposition beyond the PR thickness caused non-uniform morphology, which harmed the reproducibility of the SERS substrate (inset of Figure 4d). This result is consistent with previous reports, which indicated that an electric field that is too high hinders the formation of a homogeneous structure due to the rapid coagulation of nanoparticles and water electrolysis [35].

This phenomenon worsened over time, indicating that the differences in shape are more pronounced after 30 min of EPD. Under 5 V/cm conditions, a uniform shape formed in the PR template, and nanoparticles accelerated to connect to each other, even forming a partial film structure under 10 V/cm conditions (Figure 4e). After 60 min, most of the Au NPs were finally connected above the template, creating an Au NP film in 10 V/cm (Figure 4f). For a periodic and dense Au NP array, 5 V/cm under a 60 min condition was applied for SERS substrate fabrication.

**Figure 5:**
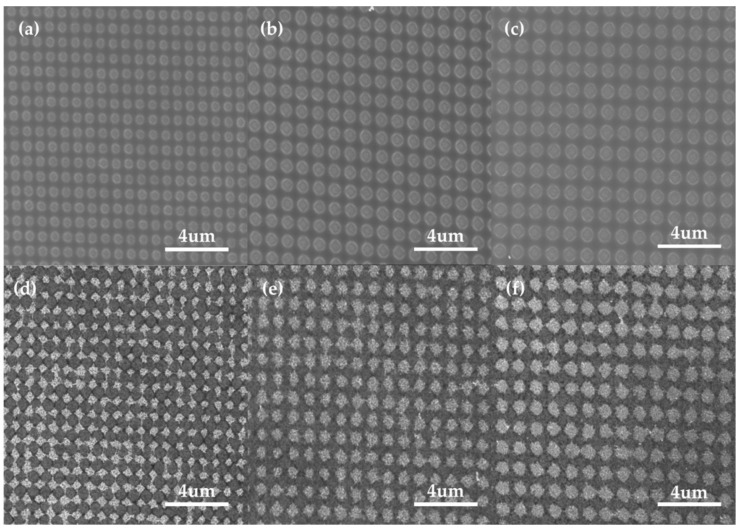
SEM image of the PR nanohole template with (**a**) 700 nm, (**b**) 900 nm, and (**c**) 1 μm period conditions, and (**d**–**f**) the substrate after Au EPD and following PR etching.

To examine the influence of the structural characteristics of the periodic Au NP array on the SERS effect, the periods and diameters of the PR nanohole template were varied, as shown in Figure 5. The angles of the incident beam and mirror were adjusted by 20°, 17°, 14°, 12°, 11°, and 10° for periods of 500 nm, 600 nm, 700 nm, 800 nm, 900 nm, and 1 μm, with the diameters of 427 nm, 530 nm, 566 nm, 599 nm, 738 nm, and 808 nm, respectively (Figure S4). The diameters of each template were also changed, and the exposure time was fixed to 4 s for the sufficient etching of PR, which revealed the ITO electrode. The surface morphologies of the substrates after the EPD of Au NP, and following PR etching, are shown in Figure 5d–f. As the diameter of the nanohole reduced from 808 nm to 427 nm, the efficient deposition for a dense array was limited to certain diameters. Au NPs were well deposited on the template, down to a 566 nm diameter with a 700 nm period. Below that condition, however, the yield of EPD considerably reduced, forming a partial deposition, such as that shown in Figure S5. This was considered to be a result of the relatively large particle size due to the collisions between particles in the EPD process. According to Derjaguin–Landau–Verwey–Overbeek (DLVO) theory [39], some moving particles, motivated by an external electric field, collide due to the difference in motion velocity, which is generated from the different sizes of the particles. In the normal EPD process, these aggregated nanoparticles move toward and deposit on the electrode, forming a homogeneous film [35]. However, in our case, these nanoparticles were restricted in terms of the size of the PR nanohole template. As a result, we found that a sufficient nanohole area is suitable for the efficient EPD of coagulated nanoparticles during the process. 

### 3.3. Raman Measurement

#### 3.3.1. SERS Activity Depending on the Different Periods

**Figure 6:**
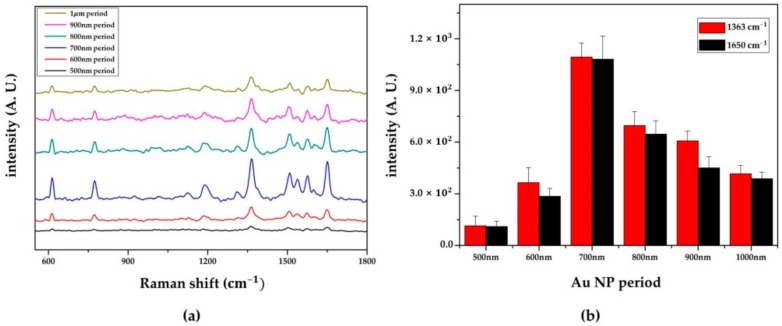
(**a**) Surface enhanced Raman spectroscopy (SERS) spectra and (**b**) the intensity bar diagram of Rhodamine 6G (R6G) 1 μM, depending on the different periods of the Au NP array.

To determine the structural condition that would enhance the highest Raman signal, the SERS spectra were measured depending on the different periods and diameter sizes of the Au NP array. The SERS spectra and the peak intensities of R6G 1 μM with different periods at 10 randomly chosen spots are shown in Figure 6a,b. The representative peaks of R6G, including 611 cm^−1^, 771 cm^−1^, 1363 cm^−1^, 1575 cm^−1^, and 1650 cm^−1^ [17,40], distinctly appeared, and the SERS performances were compared with the intensities of the peaks at 1363 and 1650 cm^−1^. When the period was reduced from 1 μm to 500 nm, the ratio of the Raman intensities changed to 1, 1.46, 1.67, 2.63, 0.88, and 0.27 at 1363 cm^−1^. For the 1650 cm^−1^ peak, the ratio changed to 1, 1.16, 1.66, 2.79, 0.74, and 0.28. Unlike the 500 to 600 nm period, which had a low EPD efficiency, we found that the SERS activity increased as the period decreased. The cause of this Raman signal augmentation may be due to the enhancement of the electromagnetic field from either the matching of the resonant wavelength, which is variable with its structural condition, or the interaction of the localized surface plasmon (LSP) with the nanostructures.

The periodic Au nanodisk structure has the ability to change the resonant wavelength according to its diameter and period [22,41,42]. If the wavelength of the incident light source and the resonant wavelength are well matched, a maximized LSPR can be obtained. In our case, however, due to the aggregation of Au NPs during the EPD process, a sufficiently large nanohole was needed to obtain an efficient EPD. When the period (700 nm) and diameter (566 nm) of the fabricated Au NP array were compared to those of previous research that simulated the resonant wavelengths with different diameters and periods [22], this kind of electromagnetic (EM) field enhancement is hard to obtain, because the predicted resonant wavelength and the wavelength of incident light have different ranges. The predicted resonant wavelength of the Au NP array was beyond the near-infrared (NIR) region, and the wavelength of the incident light source was within the visible range (514 nm). Thus, the main factor that enhanced the Raman signal was thought to be the EM field enhancement due to the increased localized surface plasmon (LSP) interactions between the Au NP arrays. As shown in previous research [43], a reduced distance between the Au nanostructures enhances the coupling of LSPR, which affects the SERS performance. The increased interactions of LSP, generated from the reduced period between the Au NP arrays, were expected to be one of the major factors that could enhance the Raman signal of the fabricated substrate (Figure 6). In addition, the hot spots, generated by the nanogaps among the adjacent Au NPs that were densely deposited in the PR nanoholes, were also thought to enhance the Raman signal. Because the distinctive peaks of 1 μM R6G were observed at a relatively long period condition of 1 μm, LSPR coupling between the Au NPs in the nanohole were expected to be a major factor enhancing the SERS activity.

As a result, the major factor that influenced the SERS effect on the fabricated substrate was the EM field enhancement from the LSPR coupling of Au NPs, both within and between the Au NP array structures.

#### 3.3.2. SERS Substrate With/Without the Patterned PR Layer

**Figure 7:**
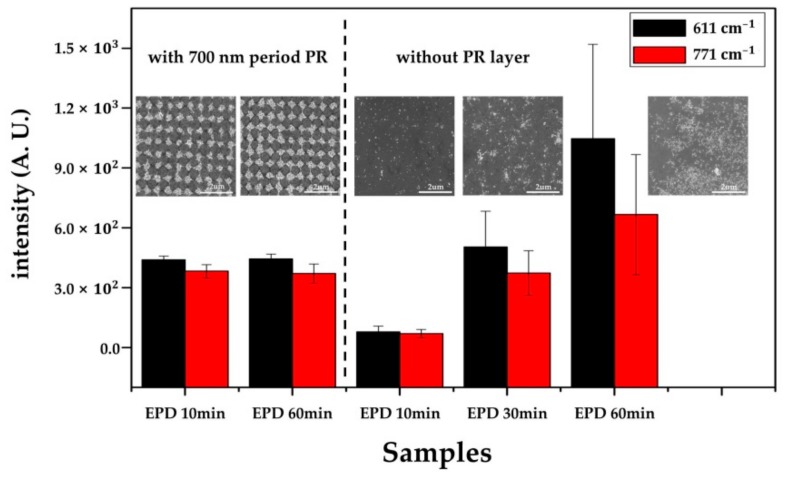
Surface morphologies and SERS performance of deposited Au NPs with and without the patterned PR layer. Insets: SEM image of the corresponding EPD conditions. To compare the SERS performance, the Raman intensity of R6G 1 μM was measured, as shown in the intensity bar diagram of each sample. We applied 5 V/cm for the EPD process.

If the Au NP layer fabricated by the normal EPD without the patterned PR layer has similar SERS performance, there is no need to use LIL to prepare the PR template. Therefore, the Raman performance was compared to verify the usefulness of the PR template layer during the EPD process. The surface morphologies of the deposited Au NPs, with and without the patterned PR layer, under the same EPD conditions (5 V/cm for 60 min), are shown in Figure 7. Whereas Au NPs were well deposited, forming a densely periodic Au NP array in the PR layer condition, a non-uniform Au NP structure was produced without the PR layer. Due to the randomly distributed Au NPs in the whole area of the electrode, different morphologies were observed in the same substrate in the absence of the PR layer between the dense and sparse NP structures. As the uniformity of the surface structure affects the deviation of the Raman signal, the relative standard deviations (RSD) of the intensities were expected to have distinctive differences between the substrates with and without the patterned PR layer. 

To compare SERS performance with the PR template layer, the intensities of 1 μM R6G at 611 and 771 cm^−1^ were evaluated. As shown in the intensity bar diagram of the EPD 60 min condition with and without the PR layer, the SERS substrate without the PR template had intensities about 2.39 times and 1.76 times stronger than the 700 nm period PR condition at 611 and 771 cm^−1^, respectively. These results might have been caused by the strong LSPR coupling of close-packed Au NPs within the laser spot size. When the Raman was measured in the relatively sparse region, however, the SERS performance seriously degraded, causing poor RSDs at 10 random points of 45.3% and 45.2% at 611 and 771 cm^−1^, respectively. In contrast, the periodic Au NP array with the uniform morphology had RSDs of 4.3% and 8.6% at 611 and 771 cm^−1^, respectively, indicating a higher reproducibility than the Au NPs layer without the PR template. As reproducibility is one of the essential requirements for the practical application of SERS, the periodic Au NP array fabricated with the PR template layer is a more suitable candidate for real SERS applications.

In addition, another advantage of the proposed process is the faster fabrication of the dense Au NP structure than the normal EPD process due to the focused electric field inside the nanohole of the PR template. From the SEM images, we found that a short EPD time of 10 min could create a dense Au NP structure, which had a morphology almost similar to that under the 60 min condition. Under a normal EPD 10 min condition, a sparse deposition of Au NPs was observed. Under the same EPD conditions (5 V/cm for 10 min), the SERS intensities of the Au NP structure with the PR layer were about 5.68 times and 5.49 times stronger than those of the Au NP structure from the normal EPD, at 611 and 771 cm^−1^, respectively. The normal EPD conditions had similar intensities to the Au NP array under a 30 min condition, although they still had bad RSDs of 35.65% and 30.16% at 611 and 771 cm^−1^, respectively. From the comparison of SERS performance, more sensitive and reproducible SERS substrates were quickly fabricated using the EPD with the patterned PR layer. Therefore, we found that the EPD with the patterned PR layer was a better fabrication strategy than the normal EPD process in terms of performance and production efficiency.

#### 3.3.3. SERS Performance

**Figure 8:**
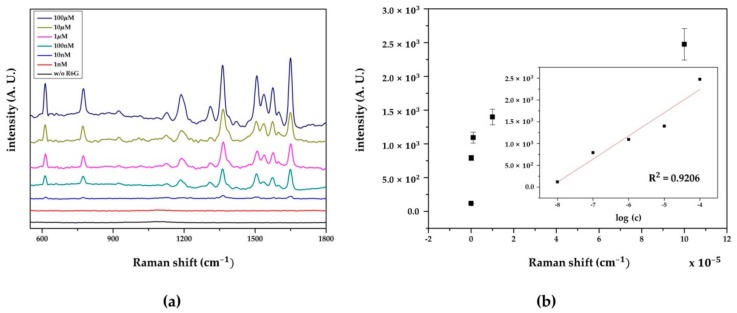
(**a**) SERS spectra and (**b**) Raman intensities of R6G at different concentrations. Raman intensities at 1363 cm^−1^ were evaluated. Inset: a linear relationship between the intensities and logarithmic concentrations of R6G.

To assess the SERS performance of the fabricated Au NP array, the variation in SERS intensities with different R6G concentrations, enhancement factor (EF), and reproducibility, was examined. The Raman spectra of R6G, from 100 μM to 1nM, which were measured and averaged at 10 randomly chosen spots, are shown in Figure 8. The specific peaks of R6G appeared distinctively from 100 μM to 100 nM, and the limit of detection (LOD) was identified as 10 nM. At 1 nM, no clear peak was observed, and the Raman spectrum of ITO without R6G had no distinctive peak affecting the Raman signal of R6G. To examine the correlation of SERS intensities and the concentrations, the peak intensities at 1363 cm^−1^ were compared at different concentrations of R6G. As shown in the inset of Figure 8b, the Raman signal increased as the concentration increased, and a logarithmically linear relationship between the SERS intensities and R6G concentration was found. The coefficient of determination was calculated as 0.9206.

Based on these results, the enhancement factor (EF) of SERS was calculated using the equation EF = $I_{\mathrm{SERS}}N_{0}/I_{0}N_{\mathrm{SERS}}$, where I_0_ and N_0_ are the normal Raman intensity and the number of molecules probed in the focused laser spot of 1 mM R6G on bare glass, respectively, without the SERS effect; ISERS and NSERS are the Raman intensity and the number of molecules probed in the focused laser spot of 10 nM R6G on the fabricated Au NP array, respectively; and $N_{0}$ was calculated using $N_{0}$ = $\frac{Ah\rho }{M}$ [40,44], where A denotes the area of the laser spot, h denotes the laser penetration depth, and ρ and M denote the density and molecular weight of R6G, respectively. $N_{SERS}$ was calculated using $N_{SERS}$ = CVA/S [40,44], where C and V are the concentration and volume of the probed molecules, respectively; A denotes the area of the laser spot; and S is the sample area. The EF was calculated to be about 1.25 × 10^5^, and the details of the EF calculation are presented in Figure S6. 

**Figure 9:**
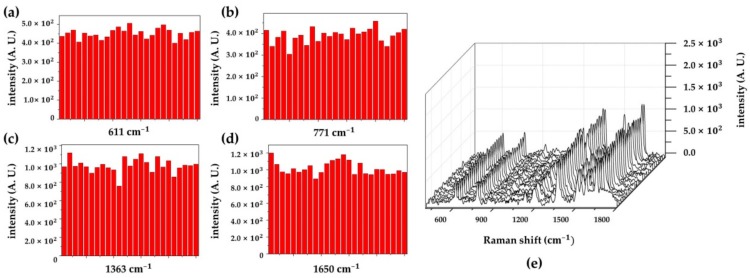
Reproducibility of the SERS signals on the Au NP array substrate. SERS intensity distribution of the Raman peaks at (**a**) 611 cm^−1^, (**b**) 771 cm^−1^, (**c**) 1363 cm^−1^, and (**d**) 1650 cm^−1^. (**e**) SERS spectra of R6G 1 μM obtained from 25 different substrates.

To examine the reproducibility of the fabricated SERS substrate, the Raman spectra of 25 different substrates were measured. As shown in Figure 9a–d, the specific peaks of 1 μM R6G, including 611, 771, 1363, and 1650 cm^−1^, were detected as distinct peaks in the SERS spectra. Their RSDs were 5.9%, 8.7%, 7.9%, and 7.8% for 611, 771, 1363, and 1650 cm^−1^, respectively. From the RSDs within 10%, we found that a reproducible SERS substrate had been fabricated. Additionally, a stability test was conducted to determine whether the fabricated SERS substrate could endure the repeated washing procedure. As shown in Figure S7, no apparent morphological changes and SERS performance degradation were observed after five repetitions of the washing process. From the SEM and Raman measurement, we confirmed that a stable Au NP array with good adhesion was fabricated.

## 4. Conclusions

A gold particle array for sensitive and reproducible SERS substrates was successfully fabricated using laser interference lithography and the electrophoretic deposition of gold nanoparticles from a pulsed laser ablation liquid process. PR templates, with various periods from 500 nm to 1 μm, were fabricated from LIL, and Au NPs with diameters of around 27 nm were physically generated from the PLAL process. The EPD of the Au NP to the PR-templated ITO electrode was conducted, and then PR etching produced a periodic Au NP array for the SERS substrate. Due to the coagulation of Au NP during the EPD process, a diameter above 566 nm was found to be suitable for efficient deposition. To determine the effect on the SERS due to its period and diameter, the Raman spectra of a R6G 1 μM solution were measured. The Au NP array under a 700 nm period condition, which had the highest EM field enhancement due to the LSPR coupling of Au NPs, both within and between the array structures, showed the highest intensity among the substrates. To verify the usefulness of the proposed fabrication strategy, the surface morphologies and the Raman performances depending on the photoresist (PR) template were compared. We found that the EPD process with the patterned PR layer was a viable fabrication strategy in terms of reproducibility and production efficiency. The performance of the fabricated Au NP SERS sensor was measured with different concentrations of R6G. Its limit of detection (LOD) was 10nM and the EF was calculated as 1.25 × 10^5^. The intensities of 1363 cm^−1^ and 1650 cm^−1^ demonstrated the good reproducibility of the fabricated SERS substrates, producing RSDs of less than 10% in the 25 different substrates. Additionally, a good adhesion stability was confirmed by the constant SERS signals during five repeated washings. The proposed method produces sensitive and reproducible SERS substrates, in a large-scale, simple, and is an inexpensive process under ambient conditions. This advantageous fabrication strategy is expected to facilitate the practical applications of SERS.

## Supplementary Materials

The following are available online at http://www.mdpi.com/1424-8220/18/11/4076/s1: Figure S1: Schematic diagram and digital image of process; Figure S2: Atomic force microscopy (AFM) measurement; Figure S3: TEM and EDS of Au NPs; Figure S4: SEM of the PR template, depending on the period; Figure S5: SEM of the EPD with a low efficiency; Figure S6: Limit of detection (LOD) selection and enhancement factor (EF) calculation; Figure S7: Raman intensities depending on the washing processes.
