# Supplementary material for: Sensitive and Reproducible Gold SERS Sensor Based on Interference Lithography and Electrophoretic Deposition
Source: Sensors (Basel). 2018 Nov 21;18(11):4076. doi: 10.3390/s18114076 (PMC6263928; doi:10.3390/s18114076)
Supplement: Supplementary file 1 [file sensors-18-04076-s001.pdf]

sensors

Supplementary Information

Sensitive and Reproducible Gold SERS Sensor Based on Interference Lithography and Electrophoretic Deposition

June Sik Hwang and Minyang Yang *

Department of Mechanical Engineering, Korea Advanced Institute of Science and Technology, Daejeon 34141, Korea; ssvsjs76@kaist.ac.kr
* Correspondence: myyang@kaist.ac.kr; Tel.: +82-42-350-3224

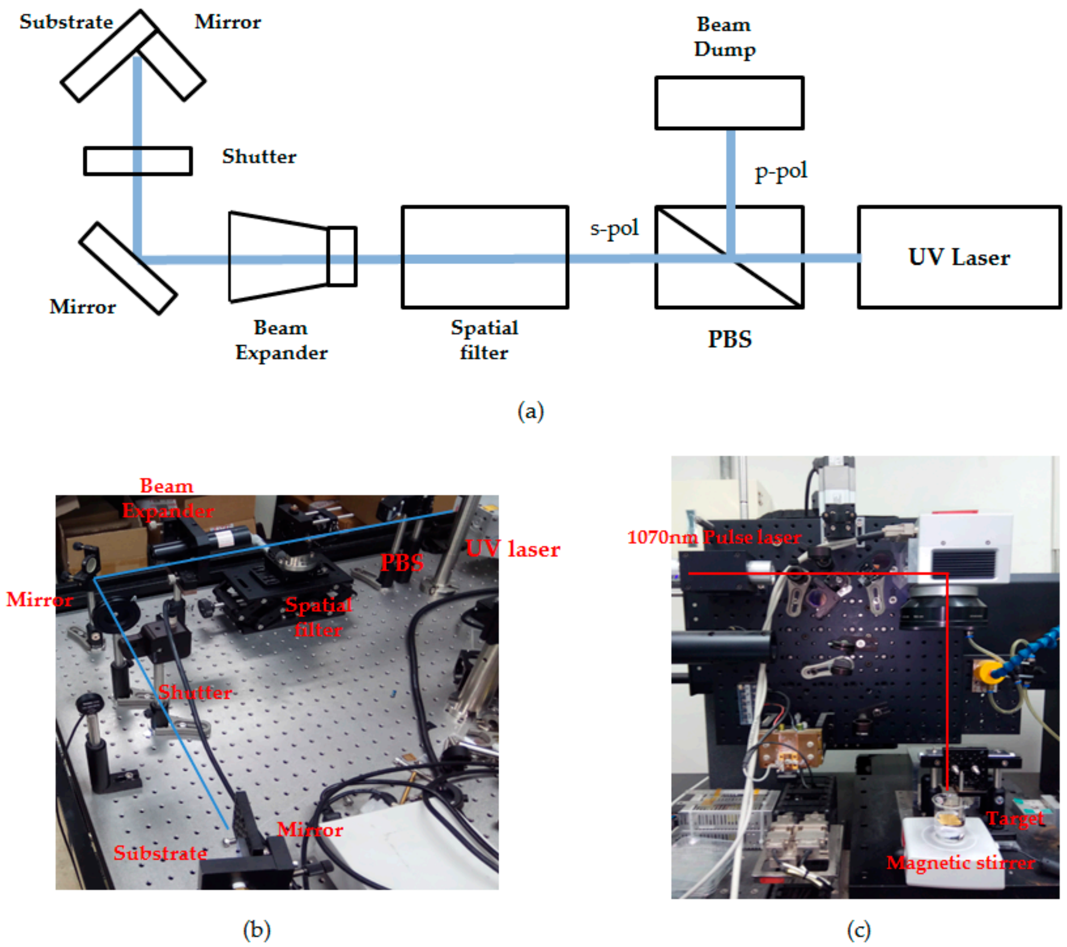

Figure S1. Schematic diagram and digital image of process: (**a**) a schematic diagram of laser interference lithography, (**b**) digital images of interference lithography and (**c**) pulsed laser ablation in liquid process.

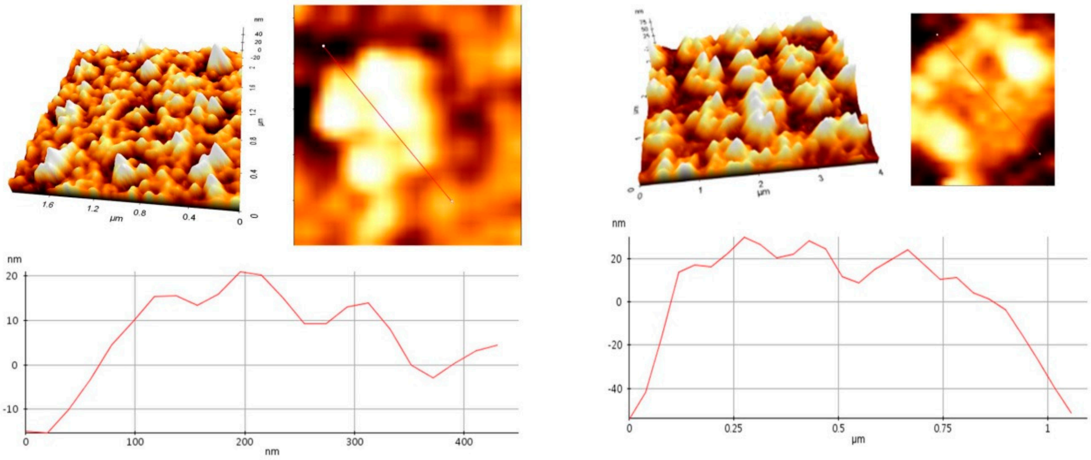

(a) (b)

Figure S2. AFM data of (**a**) 700nm period and (**b**) 900nm period Au NP array: three-dimensional measurement, line measurement, and roughness spectrum.

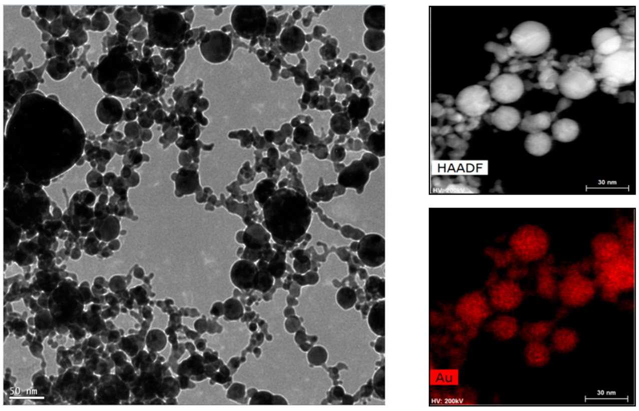

(a) (b)

Figure S3. (**a**) TEM image of Au NPs with fluence of 22.64 J cm^{-2} and (**b**) EDS of Au NP.

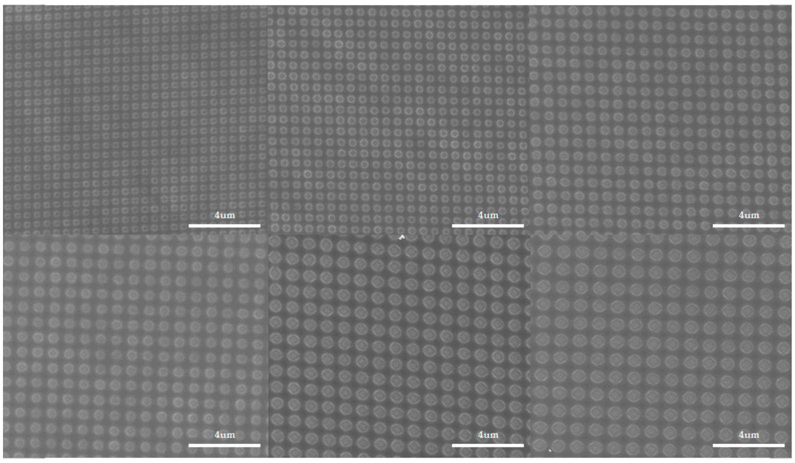

Figure S4. SEM image of PR nanohole template with 500nm, 600nm, 700nm, 800nm, 900nm and 1um period.

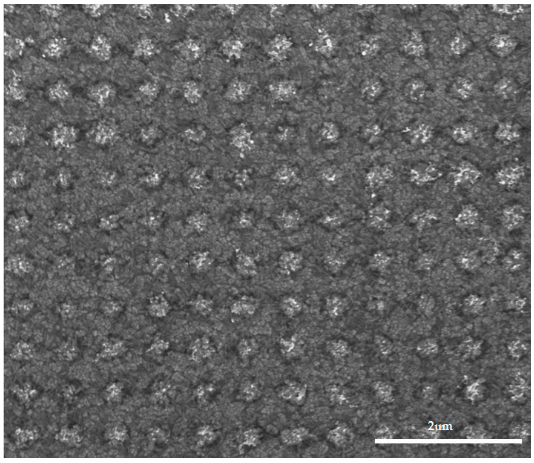

Figure S5. SEM image of 600nm period substrate after Au NP EPD and following PR etching.

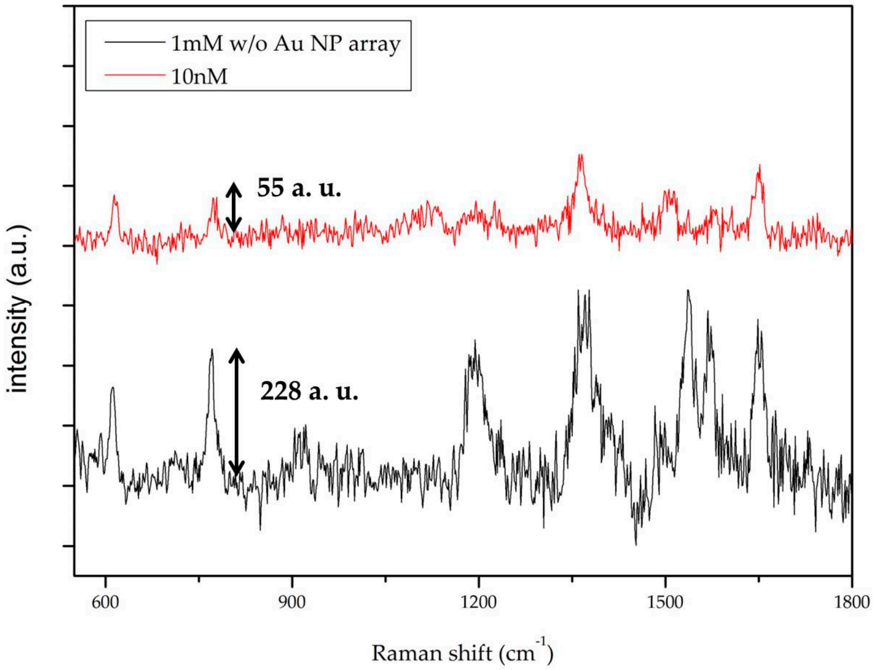

Figure S6. Raman spectra of 10nM R6G on SERS substrate and 1mM R6G on bare glass.

The measurement was conducted with to 0.35 mW of laser power in both normal Raman and SERS. 50x objective lens with 0.5 N.A. and 1um of spot size were applied for all Raman measurements. All Raman data was background-subtracted and the baseline correction was done with 5th order polynomial fitting.

For EF calculation, a normal Raman spectrum of 1mM R6G on bare glass was measured. As shown in Figure S6, specific peaks as 611 cm^{-1} , 771 cm^{-1}, 1363 cm^{-1}, 1577 $cm^{-1}$and 1650 cm^{-1} of R6G 1 mM were detected without SERS effect.

In the case of the SERS substrate, after the baseline correction, in 10 nM R6G, four specific peaks of R6G including 611 cm^{-1} , 771 cm^{-1}, 1363 cm^{-1} and 1650 cm^{-1} were detected as distinct peaks in SERS spectra. Because the smallest intensity among the distinct peaks was 55 a.u. (771 cm^{-1}) which is much higher than adjacent background signal, we selected 10 nM as the limit of detection.

The enhancement factor was calculated from EF = $(I_{SERS}N_0)$ / (I_0N_{SERS}) where N_0 and I_0 are the number of molecules probed in the focused incident laser spot and peak intensity of 1 mM R6G solution on glass substrate. N_{SERS} and I_{SERS} denote the corresponding number of molecules and the intensity of 10 nM R6G solution on Au NP array SERS substrate.

N_0 was calculated using [40,45]:

$$N_0 = Ah\rho \ /M \ = \left(\left(\frac{\pi}{4}\right) \times (10^{-6} \ m)^2\right) \times (10^{-6} \ m) \times \ 1.26 \ \text{g} \ (10^{-2} \ m)^{-3}/479.01 \ \text{g} \ mol^{-1}$$

$= 2.06 \ \times 10^{-15}$ mol

where A denotes the area of the laser spot (the spot diameter was 1 um), h (1 um) denotes the laser penetration depth, and ρ (1.26 g cm^{-3}) and M (479.01 g mol^{-1}) denote the density and molecular weight of R6G. N_0 was estimated 2.06×10^{-15} mol.

Assuming 10 nM probe molecules are entirely adsorbed and uniformly distributed on Au NP array SERS substrate,

N_{SERS} was calculated using [40,45]:

$$N_{SERS} = CVA/S =$$
$$(10^{-8} \ \text{mol} \ l^{-1}) \times (10 \times \ 10^{-6} \ l) \times (0.785 \ \text{x} \ 10^{-12} \ m^2 \ )/\left(\left(\frac{\pi}{4}\right) \times (5 \times 10^{-3} \ m)^2\right)$$

$= 4 \times \ 10^{-21}$ mol

where C (10^{-8} mol l^{-1}) and V (10 ul) are the concentration and volume of the probed molecules, respectively. A denotes the area of the laser spot (0.785 $\times 10^{-12}$ m^2) and S is the sample area $\left(\frac{\pi}{4}\right) \times (5\text{mm})^2 = 1.96 \times \ 10^{-5} \ m^2$. N_{SERS} was estimated $4 \times \ 10^{-21}$ mol.

In our measurement of 10 nM R6G, four specific peaks of R6G including 611, 771, 1363 and 1650 cm^{-1} were detected as distinct peaks in SERS spectra. The intensities of the peaks were 56.6595 a.u., 55.23902 a.u., 119.3406 a.u. and 101.6738 a.u., respectively. In normal Raman, the intensities of those peaks were 152.339 a.u., 228.2148 a.u., 219.6118 a.u. and 189.6128 a.u., respectively. Among the four peaks, the 771 cm^{-1} peak was selected for the EF calculation because it had the smallest ratio of $I_{SERS}/ \ I_0$ = 55.2902 / 228.2148 = 0.242273.

EF = $(I_{SERS}N_0)$ / (I_0N_{SERS})

$$= (I_{SERS} \ / \ I_0) \times (N_0 \ / \ N_{SERS}) = 0.242273 \times \left(\frac{2.06 \ \text{x} \ 10^{-15} \ \text{mol}}{4\text{x} \ 10^{-21} \ \text{mol}}\right) = 1.25 \times \ 10^5 \ .$$

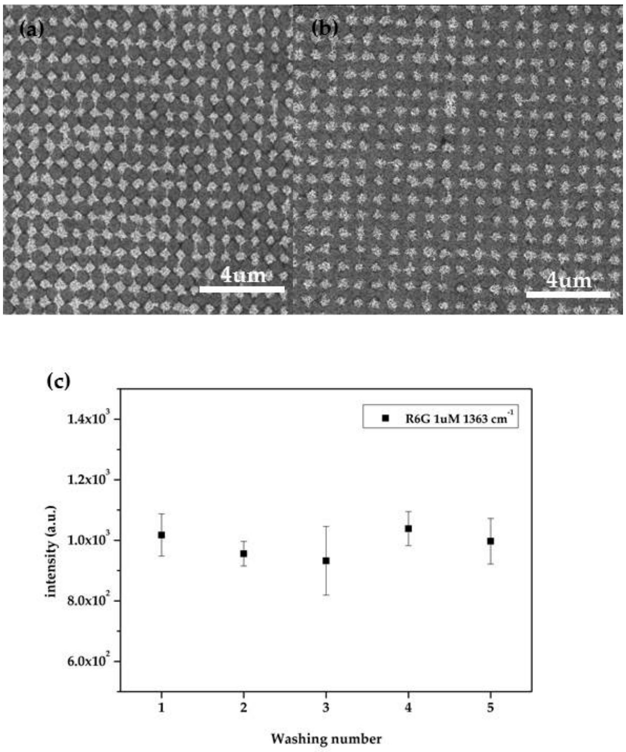

Figure S7. SEM images of Au NP array (**a**) before and (**b**) after five time washing step. (**c**) Raman intensities of R6G 1 uM at 1363 cm^{-1} depending on the washing processes.
